# Supplementary material for: Non-typhoidal Salmonella contributes to gastrointestinal infections in Morogoro: Evidence from patients attending Morogoro regional referral hospital in Tanzania
Source: PLoS Negl Trop Dis. 2024 Jun 7;18(6):e0012249. doi: 10.1371/journal.pntd.0012249 (PMC11189249; doi:10.1371/journal.pntd.0012249)
Supplement: Attachment — Submitted filename: REVIEWED_PNTD-D-24-00168_BM.pdf [file pntd.0012249.s001.pdf]

PLOS Neglected Tropical Diseases

Non-Typhoid Salmonella Contributes to Gastrointestinal infections in Morogoro; evidence from Patients Attending Morogoro Regional Referral Hospital in Tanzania

--Manuscript Draft--

**Manuscript Number:**	PNTD-D-24-00168
**Full Title:**	Non-Typhoid Salmonella Contributes to Gastrointestinal infections in Morogoro; evidence from Patients Attending Morogoro Regional Referral Hospital in Tanzania
**Short Title:**	Evidence from Patients Attending Morogoro Regional Referral Hospital in Tanzania
**Article Type:**	Research Article
**Keywords:**	Salmonella spp.; Food Safety; Antimicrobials; Foodborne Infections; PCR
**Abstract:**	Introduction<br><br>Salmonella is one of the most common causes of food-borne outbreaks and infection worldwide. Non-typhoid Salmonella (NTS) infections are common and remain a significant public health problem among important bacterial foodborne diseases. The current study aimed to establish the Non typhoid Salmonella infection and antimicrobial resistance status among selected patients at Morogoro Regional Referral Hospital (MRRH), Morogoro Region, Tanzania, to inform clinical care management and public health interventions.<br><br>Methodology<br><br>A cross-sectional study was conducted using medical records and samples were collected from hospitalised and outpatients between October and December 2021. A total of 153 participants were enrolled in the study and 132 consented to being sampled. The collected samples were analysed using standard microbiological techniques. The isolates were subjected to molecular genotyping, where Polymerase Chain Reaction (PCR) was performed targeting the 16S rDNA gene. PCR products were then submitted for sequencing to establish phylogenetic relatedness.<br><br>Results<br><br>The phylogenetic analysis identified two Salmonella seroovers; Salmonella Enteritidis and Salmonella Typhimurium. The isolates were from four adults and seven children patients. The isolates were tested against six antimicrobial agents: tetracycline, trimethoprim, gentamycin, ciprofloxacin, ampicillin and cefotaxime. Further antimicrobial assays were performed by screening 10 antimicrobial resistance genes using PCR. Overall, the highest resistance was observed in ampicillin (100%), whereas the lowest resistance was recorded for ciprofloxacin and gentamicin (9.1%). In addition, four (36.4%) of the isolates were resistant to cefotaxime and three (27.3%) to tetracycline and trimethoprim. The isolates also exhibit the presence of resistance genes for sulfamethoxazole 1&2, tetracycline (tet) A&B, Beta-lactamase CTXM, Beta-lactamase TEM, Beta-lactamase SHV, Gentamycine, Acra and acc3-1 in different occurrences. In conclusion, the overall prevalence of Salmonella species in Morogoro region was 8.3% (11/132) with Salmonella Enteritidis and Salmonella Typhimurium being the only serovars detected from adults and children stool samples.<br><br>Conclusion<br><br>Our investigation showed that both children and adults had been exposed to Salmonella spp. However, the occurrence of NTS was higher in children 5.3% (7/132) than in adults 3.0% (4/132). To stop zoonotic infections and the development of antimicrobial resistance in the community, this calls for Infection Prevention and Control (IPC) and stewardship programmes.
**Additional Information:**	
**Question**	**Response**

Powered by Editorial Manager® and ProduXion Manager® from Aries Systems Corporation

**Government Employee**<br><br>Are you or any of the contributing authors an employee of the United States government?<br><br>Manuscripts authored by one or more US Government employees are not copyrighted, but are licensed under a CC0 Public Domain Dedication, which allows unlimited distribution and reuse of the article for any lawful purpose. This is a legal requirement for US Government employees.<br><br>This will be typeset if the manuscript is accepted for publication.	No - No authors are employees of the U.S. government.
**Financial Disclosure**<br><br>Enter a financial disclosure statement that describes the sources of funding for the work included in this submission. Review the submission guidelines for detailed requirements. View published research articles from *PLOS Neglected Tropical Diseases* for specific examples.<br><br>This statement is required for submission and **will appear in the published article** if the submission is accepted. Please make sure it is accurate.<br><br>**Funded studies**<br>Enter a statement with the following details:<br>• Initials of the authors who received each award<br>• Grant numbers awarded to each author<br>• The full name of each funder<br>• URL of each funder website<br>• Did the sponsors or funders play any role in the study design, data collection and analysis, decision to publish, or preparation of the manuscript?<br><br>Did you receive funding for this work?	The author(s) received no specific funding for this work.

**Competing Interests**<br><br>On behalf of all authors, disclose any competing interests that could be perceived to bias this work.<br><br>This statement will be typeset if the manuscript is accepted for publication.<br><br>Review the instructions link below and PLOS NTDs' competing interests policy to determine what information must be disclosed at submission.	The authors declare that they have no competing interests
**Data Availability**<br><br>Provide a **Data Availability Statement** in the box below. This statement should detail where the data used in this submission can be accessed. This statement will be typeset if the manuscript is accepted for publication.<br><br>Before publication, authors are required to make all data underlying their findings fully available, without restriction. Review our PLOS Data Policy page for detailed information on this policy. Instructions for writing your Data Availability statement can be accessed via the Instructions link below.	All data generated or analysed during this study are included in this article

Powered by Editorial Manager® and ProduXion Manager® from Aries Systems Corporation

Click here to access/download;Manuscript;ANACLETA PAPER-New Version.docx

Non-Typhoid *Salmonella* Contributes to Gastrointestinal infections in Morogoro; evidence from Patients Attending Morogoro Regional Referral Hospital in Tanzania.

Anacleta Cuco[a], Ernatus Mkupasi[a], Alexanda Mzula[b], Robinson Mdegela[a]

[a] Department of Public Health and Veterinary Medicine, College of Veterinary Medicine and Biomedical Sciences, Sokoine University of Agriculture, P.O. Box 3015, Chuo Kikuu, Morogoro, Tanzania

[b] Department of Microbiology, Parasitology and Biotechnology, College of Veterinary Medicine and Biomedical Sciences, Sokoine University of Agriculture, P.O. Box 3015, Chuo Kikuu, Morogoro, Tanzania

***Correspondence author:**

Alexanda Mzula: amzula@sua.ac.tz

Co-authors' email addresses:

Robinson Mdegela: mdegela@sua.ac.tz

Ernatus Mkupasi: emkupasi@sua.ac.tz

anacletajaime@gmail.com

Abstract

Introduction

Salmonella is one of the most common causes of food-borne outbreaks and infection worldwide. Non-typhoid *Salmonella* (NTS) infections are common and remain a significant public health problem among important bacterial foodborne diseases. The

current study aimed to establish the Non typhoid *Salmonella* infection and antimicrobial resistance status among selected patients at Morogoro Regional Referral Hospital (MRRH), Morogoro Region, Tanzania, to inform clinical care management and public health interventions.

Methodology

A cross-sectional study was conducted using medical records and samples were collected from hospitalised and outpatients between October and December 2021. A total of 153 participants were enrolled in the study and 132 consented to being sampled. The collected samples were analysed using standard microbiological techniques. The isolates were subjected to molecular genotyping, where Polymerase Chain Reaction (PCR) was performed targeting the 16S rDNA gene. PCR products were then submitted for sequencing to establish phylogenetic relatedness.

Results

The phylogenetic analysis identified two *Salmonella* seroovers; *Salmonella* Enteritidis and *Salmonella* Typhimurium. The isolates were from four adults and seven children patients. The isolates were tested against six antimicrobial agents: tetracycline, trimethoprim, gentamycin, ciprofloxacin, ampicillin and cefotaxime. Further antimicrobial assays were performed by screening 10 antimicrobial resistance genes using PCR. Overall, the highest resistance was observed in ampicillin (100%), whereas the lowest resistance was recorded for ciprofloxacin and gentamicin (9.1%). In addition, four (36.4%) of the isolates were resistant to cefotaxime and three (27.3%) to tetracycline and trimethoprim. The isolates also exhibit the presence of resistance genes for *sulfamethoxazole 1&2, tetracycline (tet) A&B, Beta-lactamase* $_{CTXM}$*, Beta-lactamase* $_{TEM}$*, Beta-lactamase* $_{SHV}$*, Gentamycine, Acra and acc3-1* in different occurrences. In conclusion, the overall prevalence of *Salmonella* species in Morogoro region was 8.3%

(11/132) with *Salmonella* Enteritidis *and Salmonella* Typhimurium being the only serovars detected from adults and children stool samples.

Conclusion

Our investigation showed that both children and adults had been exposed to *Salmonella* spp. However, the occurrence of NTS was higher in children 5.3% (7/132) than in adults 3.0% (4/132). To stop zoonotic infections and the development of antimicrobial resistance in the community, this calls for Infection Prevention and Control (IPC) and stewardship programmes.

Keywords: *Salmonella* spp., Food Safety, Antimicrobials, Foodborne Infections, PCR.

1. INTRODUCTION

Salmonella infection is a major public health challenge of worldwide concern in both industrialised and developing countries and has contributed to an increased economic burden on health systems [1]. Non-typhoid *Salmonella* is a common disease in developing countries, particularly in sub-Saharan Africa, due to problems of poor sanitation, low hygiene, and lack of access to safe water and food, contributing to around 9% of deaths in children under 5 years of age [2]. Most of the *Salmonella* spp. infected individuals present diarrhoea, high fever with prostration, and stomach cramps. Symptoms typically appear 6 hours to 6 days after exposure and last 4 to 7 days [1]. Antimicrobials are only used to treat people with severe illnesses with life-threatening symptoms[3]. In severe cases, hospitalisation may be required. Every year, approximately 600 million people, or nearly one-tenth of the global population, become ill, and 420 000 die, resulting in the loss of 33 million disability-adjusted life years (DALYs), accounting for 22.2% of DALYs due to diarrhoea[4].

According to the White-Kauffmann-Le Minor scheme [5], *Salmonella has over 2600 serotypes, with the majority (approximately 1600 serotypes) belonging to the subspecies enterica*. Though many serotypes (over 200) have been identified as pathogenic agents in humans, the two most common serotypes causing human salmonellosis are *Salmonella* Enteritidis (*S.* Enteritidis) and *Salmonella* Typhimurium (*S.* Typhimurium) [6]. Both Serotypes (*S.* Typhimurium and *S.* Enteritidis) produce the symptoms of gastroenteritis and bloody diarrhoea [7]. Therefore, many strain types make it challenging to diagnose the condition rapidly and accurately [8]. In Tanzania, there few are reports of the occurrence of *Salmonella* spp. infection in both humans and animals [9], [10] and [11]. *Salmonella* infection in Tanzania, concretely in Morogoro, may be higher than expected.

Healthcare services in low- and middle-income countries are facing significant challenges due to antimicrobial multi-drug resistance, which is increasing rapidly due increased global human mobility and irrational use of antimicrobials in the health and livestock sectors [12]. Human infections with multi-resistant bacterial strains, including *Salmonella*, can result in prolonged hospitalisation and increased mortality, especially in children with invasive infections [13]. Diarrhoeic cases in patients admitted to Morogoro referral hospital are common, but the aetiologies were not well elucidated. The contribution of NTS was not well known; hence, it was often goes unnoticed, despite their disastrous effects in terms of human suffering and economic costs. Moreover, before this study, limited evidence existed on the incidence of NTS gastroenteritis in MRRH, Tanzania. Therefore, this study aimed at establishing the status of NTS and its antimicrobial profile at MRRH, Tanzania to inform its existence and treatment consideration.

Estimating the occurence of NTS among diarrhoeic hospitalised and outpatients in MRRH is paramount for planning strategies and developing preventive and control strategies to reduce admissions caused by diarrhoeic cases. In addition, the antimicrobial susceptibility profiles of the isolated NTS assessed will guide updating treatment guidelines in public health interventions.

2. MATERIALS AND METHODS

2.1 Description of the Study Area

The study was conducted in MRRH from October 2021 to December 2021. Morogoro town is the headquarters of Morogoro region, with a population of 986,678 in 2022 [14], located in the eastern part of Tanzania, 196 kilometres (122 mi) west of Dar

es Salaam. Morogoro town lies at the base of the Uluguru Mountains [15]. Morogoro residents receive health services from health institutions owned by the government and private sectors.

The study area was chosen based on the more significant number of patients attending the facility in the region. Hence, patients attending the MRRH presenting with signs suggestive of *Salmonella* spp. infectious such as stomached symptoms and gastroenteritis were recruited. The participants were characterised by their age, gender, marital status and occupation. The 132 stool samples from patients with gastrointestinal problems without diarrhoea as the first symptom admitted at the hospital and outpatient who went to the hospital complaining about diarrhoea originating in Morogoro were analysed. In this study, age groups were classified as follows; children under five years old, children (5-8 years), teenagers (13-19 years), younger (20-21 years), adults (22-60years) and elders (above 60 years).

2.2 Sampling and Patient recruitment

Purposive sampling based on clinical signs and symtoms was used to recruit participant in this study. Participants from the four hospital wards, recovery wards (male and female), surgical wards (male and female), maternity ward (females only), laboratory and outpatients were selected for recruiting patients with assistance from the clinician depending on clinical presentation and their willingness to participate in the study. Study participants were informed about the overall objective of the study, the voluntary participation, and their rights to withdraw from the study. Recruitment was subject to obtaining a written informed consent. A total of 153 patients were interviewed but only 132 conseted to collect samples from them. The respondents were divided into two groups

of children and adults, where for children, two subgroups were under five years old and above five years up to 13. Adults were further divided into four subgroups; teenagers (13-19), youths (20-21), adults (22-60) and elders (60+).

2.3 Ethical Consideration

In the present study, ethical clearance was granted by the National Institute of Medical Research, Dar es Salaam, Tanzania (Ref No.: NIMR*/HP/R.8a/Vol.IX/3808*-14th October 2021). Permission to carry out the study was also sought from the regional health management team (RHMT) (DC.65/245/01 - 18th September 2021), the Morogoro region district office and the management of the MRRH. Ethical approval for the study was given by the ethical committees of Sokoine University of Agriculture, Tanzania, with reference No SUA/AMD/R.1/8/763, approved on 18th October 2021. All study participants were informed about the study objectives and the procedures involved at enrollment participation. Before collecting fecal specimens, signed informed consent was obtained from each adult individual. Additionally, the parent or legal guardian signed a written consent form for minors under 18 years of age.

2.4 Inclusion and Exclusion Criteria

Patients with any of the main signs/symptoms suggestive of *Salmonella* spp. Infection, including abdominal pain, diarrhoea and blood in stool, were enrolled for sample collection. Patients who contracted gastroenteritis of any cause after admission and patients who did not have gastrointestinal alterations were excluded from the study.

2.5 Stool Sample Collection

Stool samples were collected from outpatients and hospitalised patients attending MRRH during the study period. The stool samples were collected from patients admitted in the

adult male recovery ward, adult female recovery ward, infectious pediatric ward, general pediatric ward, maternity ward and the laboratory. Screw-caped clean plastic containers were used for the collection of stool samples. The samples were transported to Microbiology Laboratory at the College of Veterinary Medicine and Biomedical Sciences, SUA, in an icebox within 3-4 hours of collection. They were processed upon arrival within a maximum of two hours.

2.6 Conventional identification of *Salmonella* spp.

In the laboratory, stools were enriched in peptone-buffered water and inoculated in Selenite Fecal Broth (SFB) (Becton-Dickinson-USA) overnight. Briefly, 1 g of stool sample was enriched in 9 ml of buffered peptone water (OXOID, UK) and inoculated in Selenite Fecal Broth (SFB) (Becton-Dickinson-USA), and incubated overnight for 24 h at 37°C. The enriched samples were cultured in Salmonella-Shigella Agar (SSA) and Xylose-Lysine-Deoxycholate (XLD) agar, according to ISO 6579 and ISO 21567 [16]. All suspected *Salmonella* spp. colonies were picked and then subcultured MacConkey and blood agar and incubated at 37ºC for 18- 24 hours. The bacteria grown were studied for their micro-morphological characteristics, while Gram stains were performed to investigate their micro-morphological characteristics as well. Conventional biochemical tests which involve several sugars, such as Triple Sugar Iron (TSI) and enzymatic tests were performed to identify the *Salmonella* spp presumptively. Both slide agglutination using polyvalent antisera (Poly A-E+Vi from SSI, Denmark) and Minibact-E biochemical tests (SSI, Denmark) were used to confirm isolates. The WHO National Salmonella and Shigella Center, Institute of Health in Bangkok, Thailand, used serotyping to identify specific serovars [17]. Phage typing was done for *S.* Typhimurium (5 isolates) and *S.* Enteritidis (6 isolates) according to the scheme defined by the PHLS Colindale London

at the OIE-National reference laboratory for *Salmonella*, Instituto Zooprofilattico Sperimentale delle Venezie, Italy.

2.7 Molecular Confirmation of *Salmonella* spp.

DNA Extraction

According to Carriero et al. (2016) [18], the thermal extraction technique was used to isolate the genomic DNA. A few colonies were briefly emulsified in 1.0 mL of nuclease-free water (provided by Inqaba Biotech, Hatfield, South Africa), washed by vortexing, and re-suspended in nuclease-free water. These steps were completed by boiling in a water bath at 95^0C for 5 minutes, and then the colonies were immediately placed in ice for 5 minutes. After repeating this technique, the suspension was centrifuged at 10,000 g for 10 minutes. The total genomic DNA was spectrophotometrically quantified using NanoDrop TM Lite Spectrophotometer (Thermo Scientific, Waltham, and U.S.A) and stored at -20^0C until further use [18].

PCR for the 16S rRNA gene

The amplification of the 1500bp fragment of the 16S rRNA gene was conducted by PCR using the universal bacterial primers for 16S rRNA 9F5’-GAGTTTGATCCTGGCTCAG-3’, 1542R5’-AGAAAGGAGGTGATCCAGCC-3’ in 20µl reaction mixture: PCR master mix (Bioneer, South Korea) constituted of 18µl of the reaction mix and 2µl of a DNA sample [19]. The PCR protocol was set as follows: denaturation at 94°C for 30 seconds, annealing at 58°C for 30 seconds, elongation at 72°C for 2 minutes, all these temperatures undergone 35 cycles and final extension at 72°C for 10 minutes. The post-PCR products were run in the gel electrophoresis using 1% agarose gel and 1xTE buffer for 45 minutes at 100 V. The 16S rRNA post-PCR products were sequenced using Sanger sequencing technology (ABI sequencer) at Macrogen Europe,

Netherlands. Nuclease-free water was used as a negative control, and DNA extracted from *Salmonella* Typhimurium ATCC® 13311™ (KWIK-STIK™, Minnesota, USA) was used as a positive control.

2.8 Antimicrobial Susceptibility Testing

Utilising the Kirby-Bauer's disc diffusion method in accordance with the clinical and laboratory standards institute guidelines, antimicrobial susceptibility testing was carried out [20]. The tops of well-defined colonies were touched with a sterile loop and then transferred to a tube of normal saline. The colony was emulsified on the inside of the tube near the side to prevent the colony's cells from clumping together. A 0.5 McFarland standard was used as the inoculum standard, which equates to roughly 106 CFU/mL. Muller Hinton agar plates (OXOID, UK) were used for the inoculation of *Salmonella* bacteria, which were then allowed to dry for 15 minutes. The six chosen antimicrobial disks were placed on the plates, and finally, the plates were incubated at 35ºC for 24 hours. A sterile cotton swab was dipped into the inoculum, swirled several times, and then pressed firmly against the inside wall of the tube above the fluid level to remove additional inoculum from the swab. Disks were applied to the agar surface using an Oxoid disk dispenser and sterile forceps. Plates were incubated in the air at room temperature (34ºC) for 18 hours with the plates inverted. The following standard antimicrobial disks (OXOID, UK) were used: trimethoprim (15 µg), cefotaxime (15µg), ampicillin (15 µg), tetracycline (15 µg), gentamicin (15 µg) and ciprofloxacin (15µg) [21]. Using callipers, the zones of inhibition in the cultures were quantified and evaluated. Interpretation of the results were recorded as sensitive (S), intermediate (I) or resistant (R) based on the National Committee for Clinical and Laboratory Standards Institute criteria [21]. To ensure the testing performance of the effectiveness of antimicrobial discs and the caliber of the media, the standard reference strain of *Escherichia coli* (ATCC-25922) was

utilised. The resistant group also comprised intermediate resistant strains. Non-susceptibility to three or more types of antimicrobials was the definition of multidrug resistance (MDR) [22].

2.9 Amplification of antimicrobial-resistant genes

Specific PCRs targeting 9 antimicrobial-resistant genes were performed on each pooled DNA sample using specific primers, as shown in Table 1 below. Two Duplex PCRs were conducted to detect the *Sul1* and *Sul2* genes and *bla* $_{CTX-M}$ and *bla* $_{SHV}$ genes. Singleplex PCR was performed on the rest of the antimicrobial genes. All amplifications were performed using the same regimen except for annealing temperatures, which were adjusted based on the manufacturer's recommendations (Table 1). The amplification regimen included an initial denaturation step at 95°C for 15 minutes, 30 cycles of denaturation at 94°C for 30 seconds, annealing at respective temperatures for 30 seconds, extension at 72°C for 2 minutes, followed by a final extension step at 72^{0}C for 10 minutes. *E. coli ATCC 25922* and Universal *bla* $_{CTX-M}$ were used as negative and positive controls, respectively. The PCR amplifications were electrophoresed in 1.5% agarose gel at 110V for 60 minutes and viewed in gel Tran’s illuminator machine.

Table 1: Set of primers and their respective annealing temperature used in this study to detect antimicrobial resistance gene

Antibiotic	Gene	Primer (5’ -3’)	Size	A/temp	Ref
cephalosporins	***bla*** $_{TEM}$	F-ATG AGT ATT CAA CAT TTC CG<br>R-CCA ATG CTT AAT CAG TGA GG	858	50	Tofteland *et al* (2007) [23]
		F-ATG CGT TAT ATT CGC CTG TG			Tofteland *et*

	bla $_{SHV}$	R-AGC GTT GCC AGT GCT CGA TC	862	58	*al* (2007) [23]
	Universal bla $_{CTX-M}$	F-5′-SCS ATG TGC AGY ACC AGT AA<br>R-5′-CCG CRA TAT GRT TGG TGG TG	554	58	Woodford et al.(2005) [24]<br>Malato et al. (2009) [25]
ciprofloxacin	***acrA***	(R) TGCAGAGGTTCAGTTTTGACTGTT<br>(F) CTCTCAGGCAGCTTAGCCCTAA	107	60	Velicer et al. (2006) [26]
gentamicin	***aac(3)-I***	(F) ACCTACTCCCAACATCAGCC<br>(R) ATATAGATCTCACTACGCGC	169	60	Velicer et al. (2006) [26]
tetracycline	***tet(A)***	(F) GGTTCACTCGAACGACGTCA<br>(R) CTGTCCGACAAGTTGCATGA	577	57	Woodford et al. (2009) [27]
doxycycline	***tet(B)***	(F) CCTCAGCTTCTCAACGCGTG<br>(R) GCACCTTGCTGATGACTCTT	634	56	Woodford et al. (2009) [27]
sulfonamides	***Sul1***	(F) CGGCGTGGGCTACCTGAACG<br>(R) GCCGATCGCGTGAAGTTCCG	433	69	Wang et al (2014) [28]
	Sul2	(F) GCGCTCAAGGCAGATGGCATT<br>(R) GCGTTTGATACCGGCACCCGT	293	69	

2.10 Data analysis

Data were analysed using STATA version 16 of 2018, where descriptive and inferential statistical analyses to descpribe the association of symptoms to Salmonella infection and demographic data were performed using Chi-squire. Descriptive statistics were used to present the social demographic characteristics of respondents. The data analysis was performed separately for children under 05 years old and individuals aged >05 years old. Descriptive statistical analysis was conducted using GraphPad Prism 9 software. The

sequence comparison was performed by BLASTING the sequences in www.ncbi.nlm.nih.gov/ BLAST to obtain the reference sequences from the gene bank. The sequence assembly from this study was conducted using Bioedit software version 7.2. All sequences (from this study and references) were aligned using MEGA XI software. The neighbor-joining phylogenetic tree was constructed using the same software.

3. RESULTS

3.1 Demographic characteristics of the participants

One hundred and fifty three participants involved in this study comprised of two broder groups of childen (n=65) and adults (n=88). While all the participant consented for an interview, only 132 patients consented to collect stool samples out of these patients with gastrointestinal problems admitted and outpatients at the hospital originating in Morogoro for analysis. Most of the patients (74 patients) came from rural areas, but some were from the Morogoro Municipality (58 patients). The sampled patients were in the following groups: children (n=54) and adults (n=78), further classified as teenagers (n=6), younger (n=4), adults (n=59) and elders (n=9). Of those patients, 58/132 (44%) were male and 74/132 (56%) were female. In this study, age groups were classified as follows: children (5-8 years), teenagers (9-16 years), younger (17-21 years), adults (22-60years) and elders (above 60 years).

3.2 Isolation and Molecular confirmation of *Salmonella* spp.

Of 132 samples subjected to conventional identification, 11(8.3%) were presumptively identified as *Salmonella* spp. The bacteria were non-lactose fermenters on MacConkey agar, while their colonies appeared colourless with black centres on SSA and pink with a

black centre on XLD. The microscopic findings revealed them as short rods in single, gram-negative bacteria. Molecular amplification of the 16S rDNA gene detected a PCR product band of 1500bp in gel electrophoresis (Fig 1). Sample number 11 contains multiple bands which make it appear different from the rest of the bands. These multiple bands are caused by low annealing temperature and short annealing time. But sometimes the nature of the sample itself can cause this condition to happen. Further optimization of PCR is supposed to be done to give good results. The results for phylogenetic analysis displayed two important non-typhoid *Salmonella* serotypes; *Salmonella* Enteritidis (6 isolates) and *Salmonella* Typhimurium (5 isolates) from several sequences in the gene banks (Fig 2). The sequences from this study showed a percentage identity of 97-99% during blasting with those used in phylogenetic analysis.

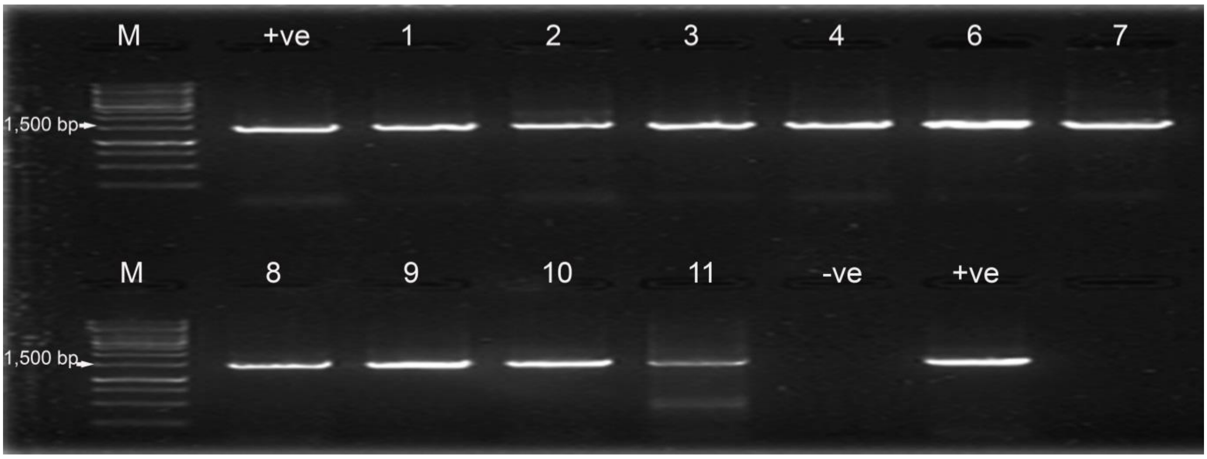

Figure 1: PCR amplification of 16S rDNA gene (1500 bp) of the 11 isolates: M = Marker DNA size Marker (1500bp), +ve = Positive control, 1-11 = Samples, -ve = Negative control.

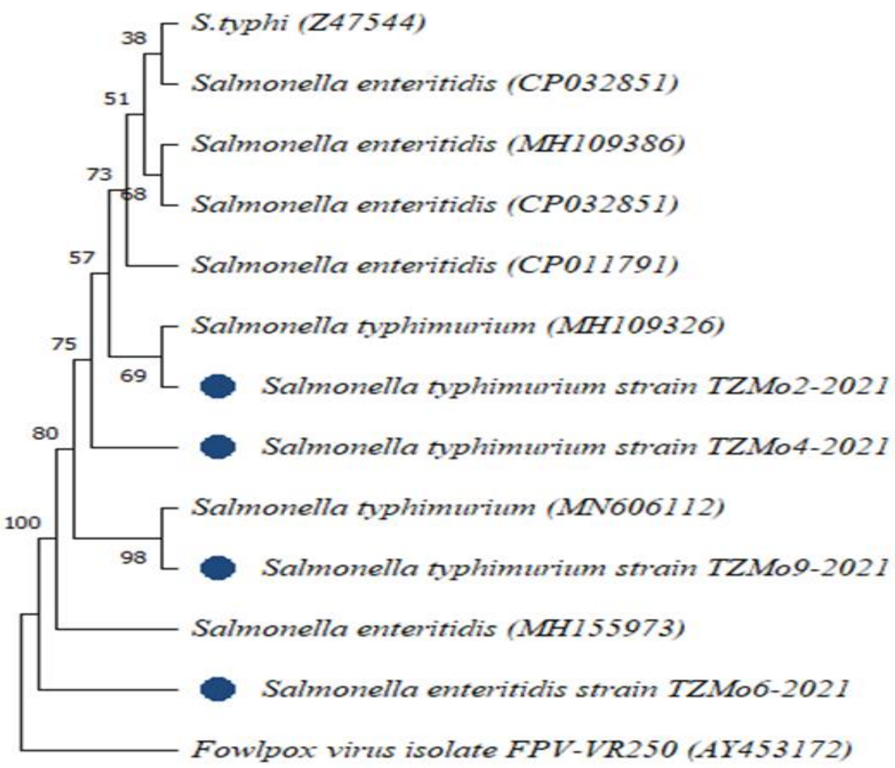

Figure 2: Phylogenetic tree for *Salmonella* serotypes from this study (blue circle) and closely related taxa from the gene bank to assess the genetic similarity of the *Salmonella* isolates from human faeces from Morogoro. The tree was generated using the Neighbor-Joining method (p-distance model), with bootstrap values expressed as percentages of 1,000 replications. *Fowlpox* virus (AY453172) served as an out-group.

3.3 The occurance of *Salmonella* spp between children and adults

A comparison of the isolation rate between adults and children showed a higher infection rate in children 5.3% (7/132) than in adults 3.0% (4/132); however, the difference (P>0.05) was not statistically significant.

3.4 Phenotypic and molecular antimicrobial susceptibility assessment of isolated *Salmonella* spp.

The results of antimicrobial susceptibility profiles of the *Salmonella* spp. isolates showed that all of the total isolates, 100% (n=11), were resistant to at least one antimicrobial. It

further showed that all of the *Salmonella* spp. isolates were resistant to ampicilin (100%, n=11). The other antimicrobial resistance occurencies were cefotaxime (36.4%, n=4), tetracycline and trimethoprim (27.3%, n=3 each). Resistance to gentamicin and ciprofloxacin was 9.1% each, as observed in Table 2.1. The results of antimicrobial susceptibility profiles of the *Salmonella* spp. isolates showed the resistance to one or more classes of antimicrobials, taking into account the *Salmonella* isolates tested with multiple resistance isolates as observed in Table 2.2. There was a marked difference in resistance between the two antimicrobials (gentamicin and ciprofloxacin) and the rest of the tested antimicrobials (*p<-0.05).* Nine different resistant genes were detected using specific primers, as shown in Table 1. The genes include the cephalosporin-resistant gene (bla_{TEM} gene, bla_{SHV} gene and universal $CTX\text{-}_{M}$ gene), ciprofloxacin-resistant gene (*arc* gene), gentamicin-resistant gene (*arc(3)-I* gene), tetracycline-resistant gene (*tetA* gene), doxycycline resistant gene (*tetB* gene) and sulfamethoxazole-trimethoprim resistant gene (*Sul 1* and *Sul 2* gene). Sulfonamide-resistant genes were highly detected (*Sal1*= 100%) compared with ciprofloxacin (0) and gentamicin (0) Table 3. The agarose gel pictures showing the corresponding band size have not been shown.

Table 2.1 Antimicrobial resistance profiles of the *Salmonella* isolates

ANTIMICROBIALS		FREQUENCY	PERCENTAGE (%)
**Tetracycline**	I	1	9.1
	R	3	27.3
	S	7	63.6
**Ampicilin**	R	11	100.0
**Cefotaxime**	I	1	9.1
	R	4	36.4
	S	6	54.5
**Gentamicin**	I	3	27.3

	R	1	9.1
	S	7	63.6
**Trimethoprim**	R	3	27.3
	S	8	72.7
**Ciprofloxacin**	I	6	54.5
	R	1	9.1
	S	4	36.4

Key: I= Intermediate resistant, R= Resistant and S= Susceptible

Table 2.2: Phenotypic and genotypic resistance proportions displayed by the identified *Salmonella* spp

**Antimicrobial category**	**Antimicrobial agent**	**Percentage phenotypic resistance (N=11)**	**Percentage Resistance genes**
Aminoglycosides	gentamicin	9.1% (n=1)	*Acc(3)-I* (n=0, 0.0%)
Extended-spectrum cephalosporins; 3rd and 4th generation cephalosporins	cefotaxime	36.4% (n=4)	Bla_{CTXM} (n= 18, 18%)<br>Bla_{SHV} (n= 2, 18%)<br>Bla_{TEM} (n= 3, 27%)
Fluoroquinolones	ciprofloxacin	9.1% (n=1)	*acrA* (n=0, 0.0%)
Folate pathway inhibitors	trimethoprim-sulphamethoxazole	27.3% (n=3)	*Sal1* (n=11, 100%)<br>*Sal2* (n=6, 54%)
Penicillins	ampicillin	100% (n=11)	Bla_{CTXM} (n= 18, 18%)<br>Bla_{SHV} (n= 2, 18%)<br>Bla_{TEM} (n= 3, 27%)
Tetracyclines	tetracycline	27.3% (n=3)	*Tet A* (n= 2, 18%)<br>*Tet B* (n=0, 0.0%)
More than two antimicrobial category	MDR	27.3% (n=3)	-

3.5 Association between *Salmonella* Symptoms and patient age category

The symptoms diagnosed included diarrhoea, blood in stool, nausea, vomiting, fever, and general pains. Results showed that nausea, vomiting and general aches and pains were significantly associated with age category ($p<0.01$). Other symptoms like diarrhoea, blood in stool and fever had no significant association with age of the patient (Table 3).

Table 3 Association between patient's age category and *Salmonella* symptoms (n=153)

Salmonella Symptoms		Patient category: Adult Count	Adult Percentage	Children Count	Children Percentage	Chi-square value (X^2)	P-value
**Diarrhoea**	**+VE**	26	29.5	29	44.6		
	**-VE**	62	70.5	36	55.4	3.687	0.055
**Σ**		88	100	65	100		
**Blood in stool**	**+VE**	2	2.3	3	4.6		
	**-VE**	86	97.7	62	95.4	0.649	0.420
**Σ**		88	100	65	100		
**Nausea (feeling sick)**	**+VE**	34	38.6	6	9.2		
	**-VE**	54	61.4	58	89.2	17.685	0.000***
**Σ**		88	100	64	100		
**Vomiting (being sick)**	**+VE**	14	15.9	23	35.4		
	**-VE**	74	84.1	41	63.1	9.414	0.009***
**Σ**		88	100	64	100		
**Feeling feverish**	**+VE**	67	76.1	41	63.1		
	**-VE**	21	23.9	24	36.9	3.071	0.800
**Σ**		88	100	65	100		
**General aches and pains**	**+VE**	62	70.5	17	26.2		
	**-VE**	23	26.1	48	73.8	34.764	0.000***
**Σ**		85	100	65	100		
	**+VE**	12	13.6	9	13.8		
**Other symptoms**	**-VE**	71	80.7	54	83.1	0.582	0.748
**Σ**		83	100	63	100		

+VE =Positive -VE =Negative * = 95% level of significance (P<0.1) *** = 95% level of significance (P<0.01)

4. Discussion

Non-typhoid *Salmonella* spp. gastroenteritis continues to be an important public health issue that often requires hospitalisation in Morogoro. This is one of the few studies exploring genetic diversity of salmonellosis in Morogoro for elders, adults, youth and young children. *Salmonella* spp. is developing resistance to many used antimicrobials, therefore, makes a relevant issue in this study.

The overall prevalence of *Salmonella* species in Morogoro region was 8.3% (11/132) (95% C.I; 5%-10.0%) with *Salmonella Enteritidis and Salmonella Typhimurium* being the only serovars detected from adults and children stool. A comparison of the isolation rate between adult and children showed comparable results in children with having higher proportion (11.6%) compared to adults (10.5%). However, the difference was not statistically significant (P>0.05).

This is concomitant to studies from Mozambique where high burden of NTS was reported among young children (*S. typhimurium* and *S. enteritidis*) [29]. Feasey et al. [30] also reported 32% compared with 54% of invasive NTS in children <15 years of age in South Africa and Malawi, respectively. In Côte d'Ivoire, the mortality rate was estimated to reach 5% from *Salmonella* infections. From studies carried out between 2005 and 2009 among isolated serotypes, NTS was prevalent [31]. It was noted from this study that *S.* enteritidis is most dominant among hospital isolates, while *S.* typhimurium serotypes were the less dominant. The previous studies conducted in Mukuru slum, an urban informal settlement in Kenya, reported *S.* Typhi as the predominant serotype followed

by *S.* typhimurium *and S.* enteritidis [32]. Previous studies in other sub-Saharan African countries also showed the dominance of *S*. typhimurium [33]. In most parts of the world, surveys have reported *S.* enteritidis and *S.* typhimurium as the major serovars found in humans [34], [35]. Different serovars of *Salmonella* spp. are known to be commonly detected in a given area at different times depending on the type of serotypes of *Salmonella* circulating in food sources in a study area [36]. In Tanzania and most other African countries, these serovars are the most frequently isolated from diarrheal diseases [37], which are confirmed by the present study's findings.

NTS resistance to the commonly used antimicrobials calls for the implementation of national surveillance systems for antimicrobial resistance and implementation of prudent antimicrobial usage, including revision of current national guidelines for antimicrobial use due to the risk of resistance. *S.* typhimurium has often been associated with multiple antimicrobial resistances [38], partly due to the emergence of *S.* typhimurium definitive phage type (DT) 104 worldwide. Strains of this phage type are resistant to ampicillin, chloramphenicol, streptomycin, sulfonamides, and tetracycline [39]. Phage typing has proven helpful for classifying *S.* enteritidis and *S.* typhimurium strains from various sources. However, the technique has shown to be inadequately selective when just one phage type has predominated, necessitating further characterisation.

The findings in this study reported a low cephalosporin resistance but high resistance to other antimicrobials among *Salmonella* strains. It was indicated that the resistance was higher to ampicillin (100%) than all other antimicrobials. The antimicrobial resistance in *Salmonella* spp. is one of the main concerns when it comes to the issue of human infection treatment [40]. Moreover, the high resistance of *Salmonella* spp. to ampicillin was also recorded by a study from Harare, where *Salmonella* spp. was resistant to ampicillin

(100%), as reported by Dinkineh et al. [41]. A similar finding was reported by Bilal et al. [42] in Pakistan. It is also important to note that, a study from Addis Ababa reported that 81.2%, 94.5%, and 75.7% of the isolates were resistant to ampicillin, tetracycline, and sulfamethoxazole-trimethoprim, respectively [43].

In contrast to the present study, 72.7%, 63.6% and 54.5% of *Salmonella* spp. isolates were shown to be susceptible to sulfamethoxazole-trimethoprim, tetracycline and cefotaxime respectively. It is also worth noting that, generally the recovered *Salmonella* spp. serovars in this study showed low resistance to cephalosporins and high resistance to other antimicrobials routinely used for the treatment of humans in Tanzania. The increased resistance towards these commonly used antimicrobials may have been contributed by misuse, incorrect diagnosis and failure to abide by treatment guidelines. This investigation identified a significant trend in the emergence of MDR in *Salmonella* typhimurium and *Salmonella* enteritidis isolates. The spectrum of resistance in NTS isolates used in this study extended further to include cefotaxime, trimethoprim-sulphamethoxazole and tetracycline, with the resistance levels agreeing with rates found in a study carried out in Thailand by Toni et al., (2018) [44]. In this study, the antimicrobial resistance gene results found that 8 isolates in adults and children contain sulfamethoxazole (*Sul 1* and *2*) resistance gene, tetracycline (*tetA*) resistance gene, Bla_{CTXM} resistance gene, Bla_{SHV} resistance gene and Bla_{TEM} resistance gene. In contrast, no any gene was detected for *tet(B*), *Acc(I-3)* and *AcrA.* These results agree with those of Hedayatianfardi et al., [45]. who found no genetic evidence for tet(B) presence in their experiments using PCR [45]. However, phenotypic results showed resistance to tetracycline, and sulfamethoxazole but only sulfamethoxazole carried the resistant gene. This is because the phenotype of most isolates is influenced by specific and non-specific

resistance mechanisms such as lower membrane permeability and high active efflux pumps [46]. Surprisingly, in the current study, the isolates carrying the *tetA* gene were phenotypically susceptible to tetracycline. Tetracycline resistance in bacteria is mediated by a group of *tet* genes through different mechanisms, including ribosomal protection and efflux pumps, [47]. On the other hand, *tet* genes have different modes of action, which, in some cases, necessitate the presence of more than one type of *tet* gene for the bacteria to fully express phenotypic antimicrobial resistance [48]. The lack of consistency in the relationship between the presence of resistance genes and phenotypic antimicrobial resistance of the isolates observed in the current study indicates the importance of accompanying the resistance gene analysis with phenotypic antimicrobial resistance testing.

Based on *Salmonella* spp. symptoms and patient category, adults and children suffering from *Salmonella* had more episodes of fever and few of them complained about visible blood in the stool. The difference, however, was statistically insignificant. The symptoms like diarrhoea, blood in stool and fever had no significant association with age of the patient in Morogoro region. Such observations are concomitant with the findings of other studies [49]. Diarrhoea was significantly related to NTS infection in Morogoro region. However, it is good to note that these clinical symptoms might be caused by other factors, including changes in diet, which can even affect faecal consistency.

5. Conclusion

In comparison to clinical and microbiological characteristics of patients with gastrointestinal problems at the hospital in Morogoro, infection rate of 8.3%NTS was

established. These results, combined with a different antimicrobials susceptibility profile, warrant further investigation into the epidemiology of NTS infections. NTS is one of the more important causes of illness in children than adults in Morogoro region, especially among children less than 5 years of age. To target the control actions to the critical points in the transmission of the pathogens to consumers, systematic surveillance of the infection sources in humans and animals, as well as the routes of the bacterial pathogens, is therefore mandatory.

6. Study limitation

This study had some limitations. Firstly, limitation on information bias. Questions for children were designed to enable parents or guardians to report on their behalf (recall bias), thus underestimate the true ORs or association of causal effect. Another limitation in this study included the use of stool samples only; multiple sampling sources, such as blood, could have created a nice ground for the broad prevalence establishment.

Abbreviations

NTS- Non-Typhoid Salmonella

MRRH- Morogoro Regional Referral Hospital

PCR- Polymerase Chain Reaction

CLSI- Clinical Laboratory Standards Institute guidelines

DALYs- Disability Adjusted Life years

SFB- Selenite Faecal Broth

SSA- Salmonella-Shigella Agar

XLD- Xylose-Lysine-Desoxycholate

ISO- International Organization for Standardization

TSI- Triple Sugar Iron

DNA- Deoxyribonucleic acid

RHMT-Regional Health Management Team

SUA- Sokoine University of Agriculture

SACIDS- Southern African Centre for Infectious Disease Surveillance

IUCEA- Inter-University Council of East Africa

TWAS- The World Academy of Sciences

Data Availability

All data generated or analysed during this study are included in this published article.

Conflicts of Interest

The authors declare that they have no competing interests.

Author's Contributions

ABJC: Aarticipated in the conception and design of the study, acquisition of data (collection of sample and laboratory analysis), analysis and interpretation of the data and writing the manuscript.

AM: Assisted in the data analysis, and molecular part and reviewed the manuscript.

EM: Approved conceptualized and designed the study and also reviewed the series of revisions on the manuscript to finalize for submission.

RHM: Designed and supervised the study, interpreted the data and helped in drafting and revising the manuscript for important intellectual content and approved the final version of the manuscript.

Acknowledgements

The authors wish to thank the Inter-University Council for East Africa (IUCEA), Southern Africa Center for Infectious Disease Surveillance (SACIDS) and The World Academy of Sciences (TWAS) for funding the study and the research and this would not have been accomplished without the assistance of SUA, MRRH and Doctors staff, and Laboratory scientists of Department of Microbiology Laboratory at the College of Veterinary Medicine and Biochemical Sciences, SUA, for analyses of samples. I appreciate the parents or guardians' contribution to this study. Additionally, I would like to thank the Morogoro Regional Referral Hospital's medical personnel, nursing staff, and administrative offices for recruiting participants. Additionally, I recognise the efforts of individuals who contributed to data collection with compensation. I am also grateful to Prof. Gerald Misinzo, who assisted in ethics application and subject recruitment for the National Institute of Medical Research, and Dr. George Alcard Rweyemamu, who assisted in Clinical Management System checking at Morogoro Regional Referral Hospital.

References

[1] F. M. Sánchez-Vargas, M. A. Abu-El-Haija, and O. G. Gómez-Duarte, “Salmonella infections: An update on epidemiology, management, and prevention,” *Travel Med. Infect. Dis.*, vol. 9, no. 6, pp. 263–277, 2011, doi: 10.1016/j.tmaid.2011.11.001.

[2] R. Das, M. A. Haque, M. J. Chisti, T. Ahmed, and A. S. G. Faruque, “Nontyphoidal Salmonella among Children under 5 Years Old in Sub-Saharan Africa and South Asia in the Global Enteric Multicenter Study,” *Am. J. Trop. Med. Hyg.*, vol. 106, no. 2, pp. 504–512, 2022, doi: 10.4269/ajtmh.21-0762.

[3] I. A. Onwuezobe, P. O. Oshun, and C. C. Odigwe, “ Antimicrobials for treating symptomatic non-typhoidal Salmonella infection ,” *Cochrane Database Syst. Rev.*, 2012, doi: 10.1002/14651858.cd001167.pub2.

[4] H. M. Chen, Y. Wang, L. H. Su, and C. H. Chiu, “Nontyphoid Salmonella infection: Microbiology, clinical features, and antimicrobial therapy,” *Pediatr. Neonatol.*, vol. 54, no. 3, pp. 147–152, 2013, doi: 10.1016/j.pedneo.2013.01.010.

[5] E. Elnekave, S. L. Hong, S. Lim, T. J. Johnson, A. Perez, and J. Alvarez, “Comparing serotyping with whole-genome sequencing for subtyping of non-typhoidal salmonella enterica: A large-scale analysis of 37 serotypes with a public health impact in the usa,” *Microb. Genomics*, vol. 6, no. 9, pp. 1–13, 2020, doi: 10.1099/mgen.0.000425.

[6] T. Eguale, “Non-typhoidal Salmonella serovars in poultry farms in central Ethiopia: Prevalence and antimicrobial resistance,” *BMC Vet. Res.*, vol. 14, no. 1, pp. 1–8, 2018, doi: 10.1186/s12917-018-1539-4.

[7] J. R. Kurtz, J. A. Goggins, and J. B. McLachlan, “Salmonella infection: Interplay between the bacteria and host immune system,” *Immunol. Lett.*, vol. 190, pp. 42–50, 2017, doi: 10.1016/j.imlet.2017.07.006.

[8] M. Bugarel *et al.*, “CRISPR-based assay for the molecular identification of highly prevalent Salmonella serotypes,” *Food Microbiol.*, vol. 71, pp. 8–16, 2018, doi: 10.1016/j.fm.2017.03.016.

[9] B. E. Ngoso, L. A. Namkinga, and G. Nkwengulila, “Molecular Characterization of Diarrheagenic Bacteria Isolated from Stool of Under-five Children in Dar Es Salaam. Tanzania,” *J. Biol. Life Sci.*, vol. 7, no. 1, p. 71, 2015, doi: 10.5296/jbls.v7i1.8929.

[10] L. A. Munuo, A. A. S. Katakweba, B. M. Lyimo, and A. P. Muhairwa, “Prevalence, characterization and antimicrobial resistance profiles of Salmonella isolates from healthy broiler and free-range chickens in Morogoro, Tanzania,” *Tanzan. J. Health Res.*, vol. 23, no. 1, pp. 1–14, 2022, doi: 10.4314/thrb.v23i1.6.

[11] O. M. Gaspary *et al.*, “Recovery and prevalence of antibiotic-resistant Salmonella from fresh goat meat in Arusha, Tanzania,” *African J. Microbiol. Res.*, vol. 10, no. 32, pp. 1315–1321, 2016, doi: 10.5897/ajmr2016.8137.

[12] K. Ilić, E. Jakovljević, and V. Škodrić-Trifunović, “Social-economic factors and irrational antibiotic use as reasons for antibiotic resistance of bacteria causing common childhood infections in primary healthcare,” *Eur. J. Pediatr.*, vol. 171, no. 5, pp. 767–777, 2012, doi: 10.1007/s00431-011-1592-5.

[13] B. Blomberg *et al.*, “Antimicrobial resistance predicts death in Tanzanian

children with bloodstream infections: a prospective cohort study," *BMC Infect. Dis.*, vol. 7, no. 1, pp. 1–14, 2007, doi: 10.1186/1471-2334-7-43.

[14] S. H. Hamisi, "Measures of Spatial Population Distribution in Morogoro Region," *Int. J. Geogr. Geol.*, vol. 11, no. 1, pp. 33–42, 2022, doi: 10.18488/10.v11i1.2920.

[15] N. Burgess, N. Doggart, and J. C. Lovett, "The Uluguru Mountains of eastern Tanzania: The effect of forest loss on biodiversity," *Oryx*, vol. 36, no. 2, pp. 140–152, 2002, doi: 10.1017/S0030605302000212.

[16] ISO, "Making food safer according to ISO methods," *Thermo-Fisher*, pp. 1–55, 2003.

[17] C. Pulsrikarn, S. Pornreongwong, C. Tribuddharat, C. Meethai, and S. Srifuengfung, "Serogroup and Serovar Distribution of Salmonella in Siriraj Hospital," *Siriraj Med J*, vol. 65, no. 6 (Suppl), pp. s34–s37, 2013.

[18] M. M. Carriero, A. A. Mendes Maia, R. L. Moro Sousa, and F. Henrique-Silva, "Characterization of a new strain of Aeromonas dhakensis isolated from diseased pacu fish (Piaractus mesopotamicus) in Brazil," *J. Fish Dis.*, vol. 39, no. 11, pp. 1285–1295, 2016, doi: 10.1111/jfd.12457.

[19] J. A. Frank, C. I. Reich, S. Sharma, J. S. Weisbaum, B. A. Wilson, and G. J. Olsen, "Critical evaluation of two primers commonly used for amplification of bacterial 16S rRNA genes," *Appl. Environ. Microbiol.*, vol. 74, no. 8, pp. 2461–2470, 2008, doi: 10.1128/AEM.02272-07.

[20] R. M. Humphries, A. N. Abbott, and J. A. Hindler, "Understanding and addressing CLSI breakpoint revisions: A primer for clinical laboratories," *J. Clin. Microbiol.*, vol. 57, no. 6, pp. 1–36, 2019, doi: 10.1128/JCM.00203-19.

[21] R. D. Walker, "Standards for antimicrobial susceptibility testing.," *Am. J. Vet. Res.*, vol. 60, no. 9, p. 1034, 1999.

[22] S. Basak, P. Singh, and M. Rajurkar, "Multidrug Resistant and Extensively Drug Resistant Bacteria: A Study," *J. Pathog.*, vol. 2016, pp. 1–5, 2016, doi: 10.1155/2016/4065603.

[23] S. Tofteland *et al.*, "Effects of phenotype and genotype on methods for detection of extended-spectrum-β-lactamase-producing clinical isolates of Escherichia coli and Klebsiella pneumoniae in Norway," *J. Clin. Microbiol.*, vol. 45, no. 1, pp. 199–205, 2007, doi: 10.1128/JCM.01319-06.

[24] N. Woodford and A. Sundsfjord, "Molecular detection of antibiotic resistance: When and where?," *J. Antimicrob. Chemother.*, vol. 56, no. 2, pp. 259–261, 2005, doi: 10.1093/jac/dki195.

[25] S. Malato, P. Fernández-Ibáñez, M. I. Maldonado, J. Blanco, and W. Gernjak, "Decontamination and disinfection of water by solar photocatalysis: Recent overview and trends," *Catal. Today*, vol. 147, no. 1, pp. 1–59, 2009, doi:

10.1016/j.cattod.2009.06.018.

[26] G. J. Velicer *et al.*, “Comprehensive mutation identification in an evolved bacterial cooperator and its cheating ancestor,” *Proc. Natl. Acad. Sci. U. S. A.*, vol. 103, no. 21, pp. 8107–8112, 2006, doi: 10.1073/pnas.0510740103.

[27] N. Woodford, A. Carattoli, E. Karisik, A. Underwood, M. J. Ellington, and D. M. Livermore, “Complete nucleotide sequences of plasmids pEK204, pEK499, and pEK516, encoding CTX-M enzymes in three major Escherichia coli lineages from the United Kingdom, all belonging to the international O25:H4-ST131 clone,” *Antimicrob. Agents Chemother.*, vol. 53, no. 10, pp. 4472–4482, 2009, doi: 10.1128/AAC.00688-09.

[28] F. H. Wang, M. Qiao, J. Q. Su, Z. Chen, X. Zhou, and Y. G. Zhu, “High throughput profiling of antibiotic resistance genes in urban park soils with reclaimed water irrigation,” *Environ. Sci. Technol.*, vol. 48, no. 16, pp. 9079–9085, 2014, doi: 10.1021/es502615e.

[29] I. Mandomando *et al.*, “Invasive non-typhoidal Salmonella in Mozambican children,” *Trop. Med. Int. Heal.*, vol. 14, no. 12, pp. 1467–1474, 2009, doi: 10.1111/j.1365-3156.2009.02399.x.

[30] N. A. Feasey *et al.*, “Typhoid fever and invasive nontyphoid salmonellosis, Malawi and South Africa,” *Emerg. Infect. Dis.*, vol. 16, no. 9, pp. 1448–1451, 2010, doi: 10.3201/eid1609.100125.

[31] Boni-Cissé C., “Serotypes and antibiotypes of Salmonella isolated at the University Teaching Hospital of Yopougon, Abidjan, Cote d’Ivoire from 2005 to 2009,” *J. Microbiol. Antimicrob.*, vol. 4, no. 2, pp. 40–44, 2012, doi: 10.5897/jma11.105.

[32] C. Hernández-Cortez, I. Palma-Martínez, L. U. Gonzalez-Avila, A. Guerrero-Mandujano, R. C. Solís, and G. Castro-Escarpulli, “Food Poisoning Caused by Bacteria (Food Toxins),” *Poisoning - From Specif. Toxic Agents to Nov. Rapid Simpl. Tech. Anal.*, 2017, doi: 10.5772/intechopen.69953.

[33] C. K. Okoro *et al.*, “Intracontinental spread of human invasive Salmonella Typhimurium pathovariants in sub-Saharan Africa,” *Nat. Genet.*, vol. 44, no. 11, pp. 1215–1221, 2012, doi: 10.1038/ng.2423.

[34] C. K. S. Saba *et al.*, “First identification of Salmonella Urbana and Salmonella Ouakam in humans in Africa,” *J. Infect. Dev. Ctries.*, vol. 7, no. 10, pp. 691–695, 2013, doi: 10.3855/jidc.3548.

[35] A. Stevens *et al.*, “Epidemiological analysis of Salmonella enterica from beef sampled in the slaughterhouse and retailers in Dakar (Senegal) using pulsed-field gel electrophoresis and antibiotic susceptibility testing,” *Int. J. Food Microbiol.*, vol. 123, no. 3, pp. 191–197, 2008, doi: 10.1016/j.ijfoodmicro.2008.01.007.

[36] R. Kebede, H. Alemayehu, G. Medhin, and T. Eguale, “Nontyphoidal Salmonella and Their Antimicrobial Susceptibility among Diarrheic Patients Attending

Private Hospitals in Addis Ababa, Ethiopia," *Biomed Res. Int.*, vol. 2021, 2021, doi: 10.1155/2021/6177741.

[37] I. J. O. Bonkoungou *et al.*, "Bacterial and viral etiology of childhood diarrhea in Ouagadougou, Burkina Faso," *BMC Pediatr.*, vol. 13, no. 1, pp. 2–7, 2013, doi: 10.1186/1471-2431-13-36.

[38] M. De Toro *et al.*, "Genetic characterization of the mechanisms of resistance to amoxicillin / clavulanate and third-generation cephalosporins in Salmonella enterica from three Spanish hospitals," pp. 173–181, 2011, doi: 10.2436/20.1501.01.146.

[39] C. Poppe, N. Smart, R. Khakhria, W. Johnson, J. Spika, and J. Prescott, "Salmonella typhimurium DT104 : A virulent and drug-resistant pathogen," vol. 39, no. September, pp. 559–565, 1998.

[40] D. Gu *et al.*, "Prevalence of Salmonella Isolates and Their Distribution Based on Whole-Genome Sequence in a Chicken Slaughterhouse in Jiangsu, China," *Front. Vet. Sci.*, vol. 7, no. February, pp. 1–10, 2020, doi: 10.3389/fvets.2020.00029.

[41] A. A. Dinkineh, K. Ameha, and M. Sissay, "Prevalence of antibiotic resistant Salmonella isolates, Entermoeba histolytica and Giardia lamblia in Harar, Eastern Ethiopia," *African J. Microbiol. Res.*, vol. 8, no. 20, pp. 2044–2053, 2014, doi: 10.5897/ajmr2014.6714.

[42] H. Bilal, M. N. Khan, T. Rehman, M. F. Hameed, and X. Yang, "Antibiotic resistance in Pakistan: a systematic review of past decade," *BMC Infect. Dis.*, vol. 21, no. 1, pp. 1–19, 2021, doi: 10.1186/s12879-021-05906-1.

[43] D. Asrat, "Shigella and Salmonella serogroups and their antibiotic susceptibility patterns in Ethiopia," vol. 14, pp. 760–767, 2008.

[44] T. Whistler *et al.*, "Epidemiology and antimicrobial resistance of invasive non-typhoidal Salmonellosis in rural Thailand from 2006-2014," *PLoS Negl. Trop. Dis.*, vol. 12, no. 8, pp. 1–19, 2018, doi: 10.1371/journal.pntd.0006718.

[45] K. Hedayatianfard, M. Akhlaghi, and H. Sharifiyazdi, "Detection of tetracycline resistance genes in bacteria isolated from fish farms using polymerase chain reaction.," *Vet. Res. forum an Int. Q. J.*, vol. 5, no. 4, pp. 269–75, 2014, [Online]. Available: http://www.ncbi.nlm.nih.gov/pubmed/25610578%0Ahttp://www.pubmedcentral.nih.gov/articlerender.fcgi?artid=PMC4299992

[46] C. Bacci, E. Boni, I. Alpigiani, E. Lanzoni, S. Bonardi, and F. Brindani, "Phenotypic and genotypic features of antibiotic resistance in Salmonella enterica isolated from chicken meat and chicken and quail carcasses," *Int. J. Food Microbiol.*, vol. 160, no. 1, pp. 16–23, 2012, doi: 10.1016/j.ijfoodmicro.2012.09.014.

[47] I. Chopra and M. Roberts, "Tetracycline Antibiotics: Mode of Action,

Applications, Molecular Biology, and Epidemiology of Bacterial Resistance," *Microbiol. Mol. Biol. Rev.*, vol. 65, no. 2, pp. 232–260, 2001, doi: 10.1128/mmbr.65.2.232-260.2001.

[48] A. C. Fluit, A. Florijn, J. Verhoef, and D. Milatovic, "Presence of tetracycline resistance determinants and susceptibility to tigecycline and minocycline," *Antimicrob. Agents Chemother.*, vol. 49, no. 4, pp. 1636–1638, 2005, doi: 10.1128/AAC.49.4.1636-1638.2005.

[49] B. R. Jackson, P. M. Griffin, D. Cole, K. A. Walsh, and S. J. Chai, "Outbreak-associated salmonella enterica serotypes and food commodities, united states, 1998-2008," *Emerg. Infect. Dis.*, vol. 19, no. 8, pp. 1239–1244, 2013, doi: 10.3201/eid1908.121511.
