# Supplementary material for: Non-typhoidal Salmonella contributes to gastrointestinal infections in Morogoro: Evidence from patients attending Morogoro regional referral hospital in Tanzania
Source: PLoS Negl Trop Dis. 2024 Jun 7;18(6):e0012249. doi: 10.1371/journal.pntd.0012249 (PMC11189249; doi:10.1371/journal.pntd.0012249)
Supplement: Attachment — Submitted filename: RESPONSES TO REVIEWERS.docx [file pntd.0012249.s002.docx]

RESPONSES TO REVIEWERS’ COMMENTS

First and foremost, we would like to thank the reviewers for their constructive comments.

We have reviewed our manuscript based on their comments, but we have also rechecked and corrected language mistakes and the proper writing of the bacteria's scientific names.

| S/N | COMMENTS | RESPONSES |
| --- | --- | --- |
|  | **REVIEWER 1** |  |
| 1 | I think this is not a proper terminology; I would suggest it to be "serovars" | The word has now been corrected to "serovars" |
| 2 | I think the authors need to tell if these patients were they outpatients or admitted to the hospital. | This was already stated in the materials and method section. It is both hospitalized and outpatient |
| 3 | In which case could the stewardship be done!? Antimicrobial use or!?? | The sentence has been qualified to explain rational antimicrobial use in health facilities and communities |
| 4 | why italicizing the sentences | Correction has been made |
| 5 | Could the age and immunity factors be associated with the high prevalence of NTS in children? | Yes, and the sentence has now been qualified to mean so |
|  |  | |
|  | REVIEWER 2 |  |
| 1 | Line 23: Salmonella spp.............(italicise Salmonella). | Done |
| 2 | Line 39: Correct spelling of serovars............. | Done |
| 3 | Intro: Cite references such as - https://doi.org/10.1099/jmm.0.022319-0. PMID: 20813852 | Done and references rearranged |
| 4 | Either use the term serovar or serotype, not both | The word serovar has now consistently been used |
| 5 | Line 117:" Change the word stomached. | Corrected |
| 6 | Line 135: error in spelling- consented? | Corrected |
| 7 | . Line 176: Should be subcultured ON...... | corrected |
| 8 | References 20-22, for AST and CLSI cite more relevant and latest references. | Corrected |
| 9 | For CLSI reference: Please clarify the year of reference, mainly for AST interpretations and MIC breakpoints. | Corrected |
| 10 | . Line 275: Correct the spelling of- broader... | Corrected |
| 11 | Discussion: Include global, regional and then local data for comparison. | Rearranged to flow from global regional and then local |
| 12 | In discussion: Names of strains needs to follow uniform way - S. Enteritidis etc. | Corrected |
| 13 | The manuscript needs to be corrected for its language by a native English speaker. | The manuscript has been reviewed for language mistakes such as Grammer |
